# Enhancing Crop Resilience: The Role of Plant Genetics, Transcription Factors, and Next-Generation Sequencing in Addressing Salt Stress

**DOI:** 10.3390/ijms252312537

**Published:** 2024-11-22

**Authors:** Akhilesh Kumar Singh, Priti Pal, Uttam Kumar Sahoo, Laxuman Sharma, Brijesh Pandey, Anand Prakash, Prakash Kumar Sarangi, Piotr Prus, Raul Pașcalău, Florin Imbrea

**Affiliations:** 1 Department of Biotechnology, School of Life Sciences, Mahatma Gandhi Central University, Motihari 845401, India; 2 Environmental Engineering, Shri Ramswaroop Memorial College of Engineering & Management, Tewariganj, Faizabad, Road, Lucknow 226028, India; 3 Department of Forestry, Mizoram University, Aizawl 796004, India; 4 Department of Horticulture, Sikkim University, Gangtok 737102, India; 5 College of Agriculture, Central Agricultural University, Imphal 795004, India; 6 Department of Agronomy, Faculty of Agriculture and Biotechnology, Bydgoszcz University of Science and Technology, Al. Prof. S. Kaliskiego 7, 85-796 Bydgoszcz, Poland; piotr.prus@pbs.edu.pl; 7 Faculty of Agriculture, University of Life Sciences “King Mihai I” from Timisoara, 300645 Timisoara, Romania; raul.pascalau@usvt.ro

**Keywords:** salt stress, salt tolerance, genetic transcription factors (TFs), next-generation sequencing (NGS), crop improvement

## Abstract

Salt stress is a major abiotic stressor that limits plant growth, development, and agricultural productivity, especially in regions with high soil salinity. With the increasing salinization of soils due to climate change, developing salt-tolerant crops has become essential for ensuring food security. This review consolidates recent advances in plant genetics, transcription factors (TFs), and next-generation sequencing (NGS) technologies that are pivotal for enhancing salt stress tolerance in crops. It highlights critical genes involved in ion homeostasis, osmotic adjustment, and stress signaling pathways, which contribute to plant resilience under saline conditions. Additionally, specific TF families, such as DREB, NAC (NAM, ATAF, and CUC), and WRKY, are explored for their roles in activating salt-responsive gene networks. By leveraging NGS technologies—including genome-wide association studies (GWASs) and RNA sequencing (RNA-seq)—this review provides insights into the complex genetic basis of salt tolerance, identifying novel genes and regulatory networks that underpin adaptive responses. Emphasizing the integration of genetic tools, TF research, and NGS, this review presents a comprehensive framework for accelerating the development of salt-tolerant crops, contributing to sustainable agriculture in saline-prone areas.

## 1. Introduction

The challenge of ensuring sufficient food production intensifies as the global population approaches a projected 10 billion by 2050 [1]. To meet the growing demand, agricultural production of cereals and livestock must increase by roughly 60%, a target complicated by the pressures of environmental stressors, particularly abiotic factors like drought, heat, and salinity [2,3,4]. These stressors disrupt key physiological and biochemical processes in plants, reducing productivity. The FAO warns that, by mid-century, salt stress alone could impact over half of the world’s arable land, heightening the food security challenge.

Salt stress, in particular, has severe economic and ecological impacts. Around 20% of irrigated farmland, or approximately 45 million hectares, is already affected by salinity, diminishing the yields of staple crops like rice, wheat, and maize, leading to estimated global economic losses exceeding USD 12 billion annually [5]. In regions highly dependent on agriculture, such as South Asia and the Middle East, this stress worsens food insecurity, driving reliance on imports and destabilizing local economies. Ecologically, salt stress contributes to land degradation and biodiversity loss. As soil salinity increases, ecosystems struggle to support various plant and animal species. Native vegetation often gives way to salt-tolerant species, resulting in reduced habitat diversity and the disruption of ecosystem services, such as nutrient cycling and water retention. In arid areas, excessive irrigation frequently causes secondary salinization, where salts accumulate due to poor drainage and insufficient water management, forcing farmers to abandon affected lands and exacerbating land degradation. For instance, in Pakistan’s Indus River Basin, extensive irrigation has led to widespread salinization, reducing arable land and threatening the livelihoods of millions who depend on agriculture [6]. These scenarios underscore the need for sustainable land and water practices to mitigate salt stress effects on agriculture and ecosystems.

Salt stress remains a major limitation to productivity, particularly in arid and semi-arid regions [5,6,7,8,9,10,11,12,13,14,15]. Currently, over 800 million hectares worldwide are affected by salinity, and this figure is expected to increase as climate change worsens the problem [16]. High salt levels in soil restrict water uptake in plants, causing osmotic stress, ion toxicity, and oxidative damage [1,4]. Salinity arises from two types of salinization: primary and secondary [17]. Primary salinization occurs naturally, through rock weathering or sea salt deposits from wind and rain [18]. Conversely, secondary salinization is largely human-induced, often through irrigation practices, which alter hydrological systems. Shifting from perennial to annual crops and using saline water, combined with poor drainage, exacerbate the problem. In regions with low rainfall, declining soil moisture further concentrates salt levels [19]. Thus, climate change, unsustainable irrigation, soil erosion, and human activities are key contributors to the growing issue of soil salinity [20].

Plants are generally categorized into two groups based on their salt tolerance: halophytes and glycophytes. Halophytes have evolved mechanisms that enable them to thrive in high-salinity environments, with some even requiring elevated salt levels for optimal growth [21]. Conversely, glycophytes are sensitive to salt, and their growth and development are significantly hindered by saline soils [22]. Notably, the majority of cultivated crops fall into the glycophyte category. For instance, crops like rice, wheat, and maize, staples for billions of people, are particularly vulnerable to salinity [2,4]. In regions such as South Asia and sub-Saharan Africa, salt stress has already reduced productivity, leading to significant food insecurity. The Indo-Gangetic Plains, home to major rice and wheat crops, have seen a decline in agricultural output due to the combined effects of waterlogging, poor drainage, and salinity, all worsened by erratic climate patterns. With food demand projected to rise by 70% by 2050 and soil salinization worsening [23,24,25,26] due to rising sea levels, erratic precipitation, and increased evaporation, addressing salt stress has become a global priority [27,28,29]. Recent advances in plant genetics, transcription factor (TF) research, and next-generation sequencing (NGS) technologies have provided promising solutions for mitigating the effects of salt stress. Intensifying research efforts have identified key genes responsible for regulating salt tolerance mechanisms in plants, including those involved in ion transport, osmotic balance, and antioxidant production [30].

Understanding the mechanisms that govern plant responses to salt stress is crucial for developing effective strategies to enhance salinity tolerance. At the core of plant responses to salinity is the Salt Overly Sensitive (SOS) pathway, which plays a pivotal role in regulating sodium homeostasis. The *SOS1* gene encodes a sodium–proton antiporter responsible for expelling sodium ions from plant cells, thereby mitigating their toxic effects. This mechanism is complemented by the *NHX1* gene, which encodes a vacuolar Na^+^/H^+^ antiporter that sequesters excess sodium into vacuoles, protecting the cytoplasm from salt-induced toxicity. Collectively, the manipulation of these key genes has facilitated the development of salt-tolerant varieties in essential crops such as rice, barley, and quinoa [31].

In addition to ion transporters, transcription factors (TFs) serve as critical regulators of gene expression in response to environmental stressors like salinity. Notable TF families, including NAC, DREB, MYB, and WRKY, have been implicated in modulating plant responses to salt stress. For instance, the overexpression of the DREB1A TF in wheat (*Triticum aestivum*) activates pathways involved in osmotic adjustment and ion homeostasis, significantly enhancing plant resilience under saline conditions [32]. Furthermore, the TF OsNAC5 in rice has been shown to improve salt tolerance by regulating stress-responsive genes and enhancing antioxidant activity, which helps prevent oxidative damage [33].

The advent of next-generation sequencing (NGS) technologies has revolutionized the study of plant responses to salt stress. RNA sequencing (RNA-Seq) allows researchers to analyze global transcriptional changes in plants exposed to salinity, revealing novel candidate genes and regulatory networks involved in salt tolerance. For example, a recent study in barley (Hordeum vulgare) used RNA-Seq to identify salt-responsive genes that enhance ion homeostasis and contribute to stress resilience, facilitating the development of robust barley cultivars [34]. Additionally, genome-wide association studies (GWASs) employing NGS have been helpful in identifying genetic loci associated with salt tolerance in crops such as quinoa, tomato, and barley, thereby aiding marker-assisted breeding for improved resilience.

Research on specific genes continues to clarify their roles in managing salt stress. In rice (*Oryza sativa*), the *OsHKT1;5* gene is crucial for sodium exclusion in roots, preventing sodium accumulation in leaves. Studies on common beans under salt stress have demonstrated that elevated NaCl levels result in decreased plant height, reduced leaf area, and fewer leaves [35]. Similar findings have been reported for soybeans subjected to salt stress, which hinders their overall growth [36]. Furthermore, observations in peas show that salt stress affects sodium distribution in roots and buds, impeding seedling growth [37]. Many legumes exhibit high sensitivity to elevated soil salinity, which adversely impacts various aspects of their development [38]. Additionally, research on mung beans has highlighted the detrimental effects of salt stress on plant growth [39].

**Figure 1:**
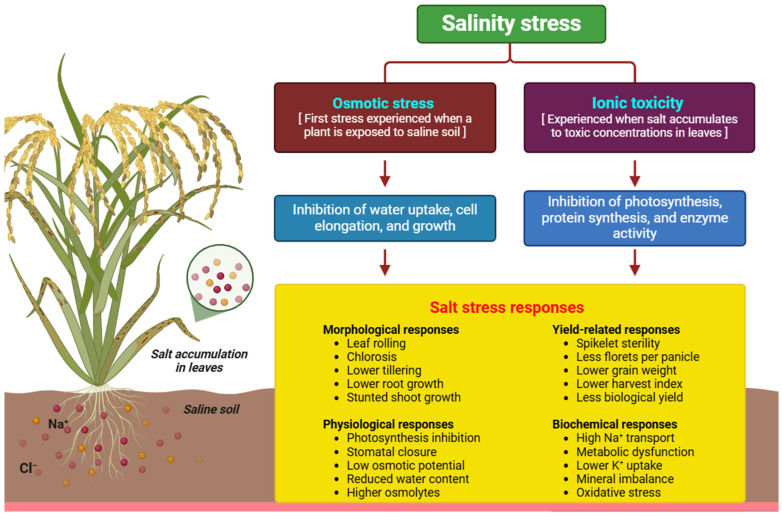
Salt stress effects on the growth and development of rice and wheat plants.

Innovative gene-editing technologies, particularly CRISPR/Cas9, have provided new avenues for developing salt-tolerant crops. Researchers have successfully manipulated genes to produce salt-tolerant rice varieties that can thrive in saline-prone regions, such as coastal areas of Bangladesh. Notable varieties, including BRRI dhan47, demonstrate resilience in high-salinity environments, significantly improving yields and food security where soil salinization has historically threatened agriculture [40,41]. In Bangladesh, where seawater intrusion exacerbates soil salinity, the introduction of these salt-tolerant rice varieties has had a transformative impact on food production and livelihoods, with yields increasing by up to 40% in saline-affected areas. The integration of plant genetics, transcription factor manipulation, and NGS technologies holds immense promise for enhancing crop resilience to salt stress. By combining conventional breeding approaches with modern genetic engineering and genome-editing techniques, researchers are developing crop varieties that are better adapted to saline environments without sacrificing yield. The CRISPR/Cas9 technology, for example, has been successfully applied to edit the *OsHKT1;5* gene in rice, resulting in plants, which exhibit enhanced salt tolerance while maintaining productivity, as shown in Figure 1 [34]. This integrated approach is essential for addressing the growing problem of soil salinization, particularly in regions most vulnerable to the effects of climate change. Salt stress poses a significant threat to global food security, but recent advances in plant genetics, transcription factors, and NGS technologies offer a pathway to mitigate its impact. By leveraging these cutting-edge tools, researchers are developing crops that are more resilient to salinity, which will be critical in sustaining agricultural productivity and meeting the demands of a growing global population. With climate change accelerating the degradation of arable land through increased soil salinization, the development of salt-tolerant crops will be indispensable in securing future food supplies. High salinity disrupts plant cellular homeostasis, leading to ion imbalance and osmotic stress, which impair growth and yield. Developing salt-tolerant crops is essential for maintaining agricultural productivity in saline environments [42]. Advances in plant genetics, molecular biology, and biotechnology have provided insights into the mechanisms of salt tolerance, offering new avenues for crop improvement.

This review explores the roles of plant genetics, transcription factors, and next-generation sequencing technologies in mitigating salt stress, emphasizing key examples and research findings, which demonstrate their impact and importance. Although previous reviews have extensively discussed salt stress mechanisms and the role of genetic factors in plant resilience [18,29,34,40,43,44,45,46,47,48,49,50,51], this review uniquely focuses on the integration of recent advancements in plant genetics, transcription factor (TF) research, and next-generation sequencing (NGS) technologies to address salt stress in a comprehensive manner. Unlike other reviews, which often examine these components in isolation, this study highlights their interconnected roles in enhancing crop resilience. For instance, by combining genome-wide association studies (GWASs) with functional genomics, researchers have pinpointed novel gene variants that enhance salt tolerance across various crop species [52], paving the way for targeted breeding programs. Additionally, advanced TF research has revealed transcriptional regulatory networks that can be harnessed to optimize ion balance, osmotic adjustment, and antioxidative defense in salt-stressed plants [53,54]. Specifically, this review emphasizes cutting-edge applications, such as the use of NGS for high-resolution gene mapping and the strategic manipulation of TFs to activate stress-response pathways. By examining these synergistic approaches, this review provides actionable insights for developing salt-tolerant crops and addresses a critical gap in the existing literature. The primary aim of this study is to offer a framework for researchers and breeders that combines genetic, transcriptional, and sequencing-based tools, thus paving the way for robust solutions in salt-stressed agricultural systems.

## 2. Salt Stress in Plants

This section addresses the challenges posed by salt stress to plant systems, a major obstacle in achieving optimal crop yields in saline environments. The discussion is organized under two key headings: Section 2.1 and Section 2.2. The first subsection examines the adverse physiological and biochemical impacts of salt stress, such as osmotic imbalance, ion toxicity, and oxidative damage, all of which lead to compromised growth and productivity. The second subsection explores plants’ inherent and engineered regulatory mechanisms for coping with salt stress and reviews innovative mitigation approaches that bolster plant resilience in salt-prone conditions.

### 2.1. Effect of Salt Stress on Plants

Salt stress is a significant abiotic factor that adversely affects plant growth, development, and agricultural yields. High levels of sodium chloride (NaCl) in the soil disrupt key physiological processes, particularly in arid and semi-arid regions where both irrigation and natural salinity contribute to the issue. Climate change exacerbates this problem through rising sea levels and shifting precipitation patterns. The understanding of salt stress’s physiological and biochemical impacts, alongside its ecological and economic ramifications, is essential for developing effective mitigation strategies. Salt stress compromises water uptake, nutrient absorption, and various metabolic functions in plants [55]. One immediate physiological effect of salt stress is osmotic stress, where high external salt concentrations lower the soil’s water potential, making water uptake by plants challenging. This results in cellular dehydration, wilting, reduced turgor pressure, and stunted growth. Biochemically, salt stress disrupts metabolic pathways, significantly affecting photosynthesis due to stomatal closure, which limits CO_2_ uptake and chlorophyll degradation under prolonged salt exposure. Additionally, toxic levels of sodium (Na^+^) and chloride (Cl^−^) ions accumulate in plant tissues, causing cellular toxicity and inhibiting enzymatic activities [56]. Salt toxicity arises from ion imbalance, osmotic stress, and oxidative damage. Elevated salinity leads to Na^+^ and Cl^−^ accumulation, disrupting essential nutrients’ balance, particularly potassium (K^+^), calcium (Ca^2+^), and magnesium (Mg^2+^). This ionic imbalance impairs crucial processes like protein synthesis and enzyme activation [57]. Sodium toxicity is particularly detrimental, as it competes with potassium for vital functions such as stomatal regulation. Glycophytic plants like rice (*Oryza sativa*) demonstrate sensitivity to Na^+^ accumulation, which interferes with K^+^ uptake, resulting in nutrient deficiencies and reduced growth [58]. The hyperosmoticf environment created by high salt concentrations further complicates water absorption, leading to diminished cell expansion and premature senescence. Some salt-tolerant species, like halophytes, mitigate osmotic stress by producing osmolytes, such as proline and glycine betaine, to maintain cell turgor and protect against dehydration. For example, barley (*Hordeum vulgare*) accumulates osmoprotectants under salt stress, aiding water balance and growth in saline environments [59].

Salt stress induces the overproduction of reactive oxygen species (ROS) such as hydrogen peroxide (H_2_O_2_) and superoxide radicals (O_2_^−^), leading to oxidative damage in plants. This exacerbates ion toxicity and osmotic stress, impairing growth. Antioxidant systems, including enzymes like superoxide dismutase (SOD) and catalase, help neutralize ROS, enhancing resilience in crops like wheat (*Triticum aestivum*) [60]. Table 1 provides a summary of the effects of salt stress on plants, highlighting both physiological and biochemical impacts.

### 2.2. Regulatory Mechanisms and Mitigation Strategies

**Figure 2:**
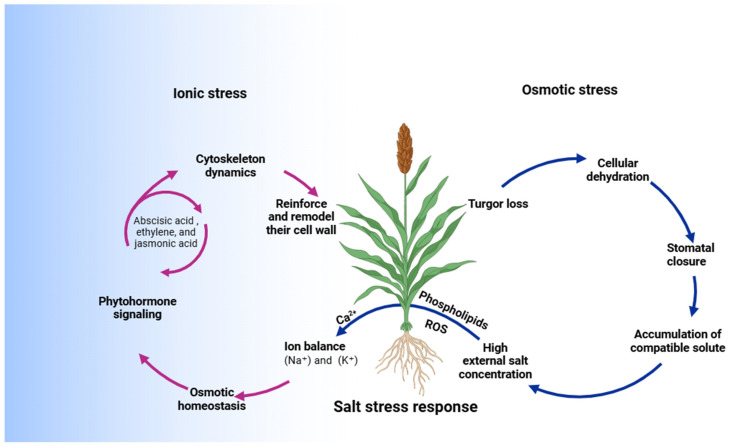
Pathways involved in salt stress response in plants: distinct mechanisms for ionic and osmotic stress. This figure illustrates the complex network of signaling pathways activated in plants under salt stress. It highlights the key roles of signaling molecules such as calcium ions (Ca^2+^), reactive oxygen species (ROS), phospholipids, and phytohormones in regulating cellular adaptations. The figure distinguishes between ionic and osmotic stress responses, showing how plants maintain ion balance, osmotic homeostasis, and cellular integrity. It also emphasizes the involvement of cytoskeletal dynamics, cell-wall modification, metabolic adjustments, and growth regulation that, together, enhance plant salt tolerance.

When plants encounter salt stress, an intricate signal transduction mechanism is activated to manage and counter the negative impacts of excess sodium ions (Na^+^) and osmotic imbalance as revealed in Figure 2. This regulatory mechanism begins with the detection of increased Na^+^ in the cell, which triggers a cascade of downstream responses involving ion homeostasis, osmotic stress management, and the detoxification of reactive oxygen species (ROS), as described previously [40,67,68,69]. The mechanism involved can be summarized as follows:

(i) Ion Sensing and Na^+^ Detection: High Na^+^ concentrations disrupt cellular ion balance, leading to toxicity. Plants respond by activating transporters that help maintain cellular ion homeostasis. One primary response is the detection of elevated Na^+^ levels, which immediately triggers pathways to reduce Na^+^ accumulation in cells, often by directing the Na^+^ ions to the vacuoles or extruding them from the cytoplasm.
(ii) Calcium (Ca^2+^) Signaling as a Secondary Messenger: In response to ion imbalance, an influx of Ca^2+^ into the cytoplasm occurs through calcium-permeable channels. Ca^2+^ acts as a crucial secondary messenger in salt stress signaling, initiating further downstream responses. The elevated Ca^2+^ levels activate specific proteins and transcription factors that control genes associated with salt tolerance, facilitating plants’ adaptive responses.
(iii) Role of Osmosensors in Calcium Regulation: Proteins such as OSCA1—a plasma membrane calcium channel—are key to initiating calcium signaling under osmotic stress. When activated by changes in osmotic pressure, OSCA1 channels allow Ca^2+^ to enter the cell, facilitating rapid signal propagation throughout the plant. Mutations in OSCA1 disrupt this calcium signaling, reducing the plant’s ability to respond effectively to salt stress.
(iv) Additional Osmosensors (KEA1/2 and KEA3): KEA1/2 and KEA3 proteins play roles in maintaining Ca^2+^ homeostasis, working alongside OSCA1 to control Ca^2+^ levels and enhance the plant’s response to osmotic stress. These osmosensors ensure that calcium signaling remains balanced, avoiding excessive Ca^2+^ levels which could be harmful, while still allowing the transmission of stress signals to initiate protective responses.
(v) Ion Transport and Na^+^ Exclusion Mechanisms: Once calcium signaling is activated, plants initiate mechanisms to manage Na^+^ levels within cells. Transporters like the Na^+^/H^+^ antiporter (SOS1) on the plasma membrane help expel Na^+^ from the cytoplasm, while other transporters, such as the NHX antiporters in the vacuolar membrane, sequester Na^+^ into vacuoles. This dual approach reduces cytoplasmic Na^+^ concentration, protecting the cell from ion toxicity and maintaining a favorable K^+^/Na^+^ ratio, essential for cellular processes.
(vi) Reactive Oxygen Species (ROS) Management: Salt stress also elevates ROS production, leading to potential oxidative damage in plant cells. Plants activate antioxidant enzymes like superoxide dismutase (SOD), catalase (CAT), and peroxidase (POD) to scavenge ROS, minimizing oxidative stress. ROS signaling also interacts with calcium signaling, reinforcing salt stress responses by regulating the transcription factors involved in stress resistance.

Together, these mechanisms constitute a highly coordinated network that enables plants to detect, respond to, and adapt to elevated salinity levels. By integrating ion transport, calcium signaling, and ROS detoxification, plants can maintain cellular integrity and functionality even under adverse saline conditions. Further, a critical component of this network is the Na^+^-gated calcium channel *MOCA1*, which plays a pivotal role in mediating Ca^2+^ signaling during ionic stress. It regulates calcium influx through its involvement in the biosynthesis of glycosyl inositol phosphorylceramide (GIPC), a monovalent cation sensor which detects Na^+^ and modulates salt stress responses. Mutations in *MOCA1* lead to hypersensitivity to salt stress. Additionally, the plasma membrane receptor-like kinase FERONIA (FER) helps maintain cell-wall integrity under salt stress by regulating Ca^2+^ signaling [70]. FER interacts with cell-wall pectin to detect stress-induced damage and works alongside cyclic nucleotide-gated ion channels (*CNGCs*) to control calcium signaling. Finally, ROS accumulation under salt stress also plays a key role in calcium signaling. The receptor kinase HPCA1 detects hydrogen peroxide (H_2_O_2_) and facilitates the Ca^2+^ influx necessary for stress responses like stomatal closure [70]. This highlights the integral role of calcium signaling in plant adaptation to salt stress.

## 3. Plant Genetics and Salt Stress Tolerance

**Figure 3:**
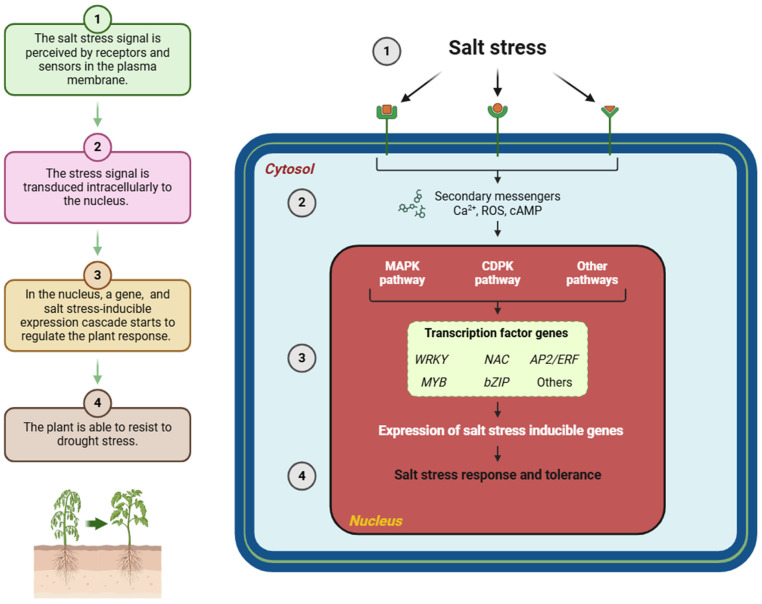
Salinity stress signaling pathways. This figure illustrates the molecular mechanisms underlying salinity stress signaling in plants. Salt stress is initially perceived by specific receptors or sensors on the plasma membrane (Step 1), which activate intracellular signaling cascades (Step 2). These cascades involve the generation of secondary messengers, such as calcium ions (Ca^2+^), reactive oxygen species (ROS), and cyclic AMP (cAMP). These molecules play crucial roles in amplifying and transmitting the salt stress signal to downstream effectors. In the cytosol, the mitogen-activated protein kinase (MAPK) pathway and calcium-dependent protein kinase (CDPK) pathway (including other pathways) are activated. These signaling pathways facilitate the phosphorylation of key proteins and transcription factors (TFs), including WRKY, NAC, DREB, MYB, and SOS, which are responsible for regulating salt stress-inducible genes (Step 3). These TFs modulate gene expression to restore ion homeostasis, regulate osmotic balance, and initiate antioxidant responses. The right panel of the figure highlights these regulatory pathways. MAPKs primarily regulate stress-responsive gene expression, while CDPKs act as calcium sensors, linking Ca^2+^ signaling to transcriptional changes. These processes culminate in the expression of salt tolerance genes, enabling the plant to adapt to saline conditions (Step 4). This mechanism ensures physiological and biochemical adjustments to mitigate salt stress impacts.

Salt stress tolerance in plants is governed by specific genetic mechanisms that regulate physiological responses to high salinity. A pivotal mechanism involves the regulation of ion transporters, which are crucial for maintaining ionic balance under saline conditions. For instance, the *NHX1* gene encodes a vacuolar Na^+^/H^+^ antiporter that plays a critical role in sequestering excess Na^+^ ions into vacuoles, thus reducing cytotoxicity in the cytoplasm. The overexpression of *AtNHX1* from *Arabidopsis thaliana* in rice (*Oryza sativa*) exemplifies this mechanism, demonstrating that enhanced Na^+^ compartmentalization leads to improved growth and higher salinity tolerance [71,72]. Figure 3 depicts the cellular responses of plants to high salinity, outlining the mechanisms and components which enable plants to adapt to saline conditions.

Another key genetic mechanism is the SOS (Salt Overly Sensitive) signaling pathway, which is essential for Na^+^ homeostasis. At the core of this pathway is the gene *SOS1*, which encodes a plasma membrane Na^+^/H^+^ exchanger. This transporter facilitates the extrusion of excess Na^+^ ions from the cell, thereby mitigating potential toxic effects. The effective functioning of *SOS1* relies on the regulatory activities of two other proteins, *SOS2* and *SOS3*. *SOS2* is a serine/threonine protein kinase, while *SOS3* acts as a calcium sensor which responds to elevated intracellular Ca^2+^ levels, a common response to salt stress [73]. The mechanism of action comprises three key components—*SOS1*, *SOS2*, and *SOS3* [73]—which collaborate to regulate Na^+^ homeostasis through the following processes:

(i) SOS1—Plasma Membrane Na^+^/H^+^ Exchanger: *SOS1* encodes a plasma membrane Na^+^/H^+^ exchanger that actively extrudes Na^+^ ions from the cytoplasm into the external environment. This is crucial for reducing Na^+^ accumulation in the cell, which can be toxic at high concentrations.
  Mechanism: *SOS1* utilizes the electrochemical gradient established by proton pumps (H^+^-ATPases) to exchange Na^+^ ions with H^+^ ions. When Na^+^ levels rise due to salt stress, SOS1 enhances the Na^+^ efflux, helping to maintain cellular ion balance and preventing Na^+^ toxicity.
(ii) SOS2—Serine/Threonine Protein Kinase: *SOS2* acts as a regulatory protein kinase that is involved in the phosphorylation of target proteins in the SOS pathway.
  Mechanism: In response to elevated salinity, *SOS2* is activated by the binding of calcium ions (Ca^2+^) that enter the cell in response to stress signals. Once activated, *SOS2* phosphorylates *SOS1*, enhancing its activity. It also phosphorylates other downstream targets that are involved in stress response, thereby integrating calcium signaling with Na^+^ homeostasis.
(iii) SOS3—Calcium-Binding Protein: *SOS3* acts as a calcium sensor that is essential for the activation of *SOS2*.
  Mechanism: Under salt stress, the increase in cytosolic calcium concentration activates *SOS3*. This activated form of *SOS3* binds to *SOS2*, facilitating its phosphorylation activity. The *SOS3-SOS2* complex plays a critical role in transducing the salinity signal, ultimately leading to the activation of *SOS1.*

Traditional breeding methods have sought to enhance salt tolerance through the selection of naturally resilient varieties, such as *Pokkali* and *Nona Bokra*. However, the complexity of salt tolerance traits often controlled by multiple genes can limit the effectiveness of these approaches [44]. In contrast, genetic engineering offers a targeted solution by introducing specific salt-tolerance genes into crops. For instance, the *OsHKT1;5* gene, which facilitates sodium exclusion from shoots, has been successfully integrated into elite rice varieties, resulting in significant increases in salt tolerance [74]. The mechanism involves the *OsHKT1;5* gene, which encodes a high-affinity potassium transporter essential for salt tolerance in rice by facilitating the exclusion of Na^+^ ions from shoots. Overexpression of *OsHKT1;5* enhances the plant’s capacity to selectively absorb potassium (K^+^) while excluding sodium (Na^+^), maintaining an optimal K^+^/Na^+^ ratio in plant tissues. This balance is crucial for cellular function, as potassium plays a key role in enzyme activation, osmotic regulation, and other physiological processes necessary for growth. Transgenic rice varieties expressing higher levels of *OsHKT1;5* have shown significant improvements in growth and yield under saline conditions, underscoring the potential of this targeted genetic approach to enhance salt tolerance and agricultural productivity in stress-prone environments [74]. One notable example of genetic engineering to improve salt tolerance is the modification of rice (*Oryza sativa*). Researchers introduced the *AtNHX1* gene from *Arabidopsis thaliana* into rice, which increased the plant’s ability to compartmentalize Na^+^ in vacuoles, thus enhancing salt tolerance [75]. Transgenic rice plants showed improved growth and yield under saline conditions, with significantly higher Na^+^ accumulation in the vacuoles and reduced Na^+^ toxicity in the cytoplasm compared to non-transgenic controls [76]. Additionally, modifications in other crops, such as tomatoes with the *SlSOS2* gene, highlight the potential of genetic engineering to develop crops better suited for saline environments [77]. The *SlSOS2* gene, similar to the *SOS2* gene in Arabidopsis, plays a crucial role in regulating Na^+^ homeostasis in tomatoes. The mechanism involves genetic modifications that upregulate *SlSOS2* expression, thereby enhancing the Na^+^/H^+^ exchange activity at the plasma membrane, akin to the function of *SOS1*. This increased exchange activity improves the plant’s capacity to extrude Na^+^ ions, allowing transgenic tomato plants to maintain normal growth and yield even under saline irrigation. Field trials have validated the effectiveness of these modifications, showing that genetically engineered tomatoes exhibit robust resilience to high salinity. Furthermore, research on wheat (*Triticum aestivum*) has demonstrated that overexpressing the *TaHKT1;5-D* gene, which modulates Na^+^ transport from roots to shoots, significantly decreases sodium accumulation in leaf tissue. This genetic modification enhances salt tolerance by promoting improved photosynthetic activity and growth under saline conditions [78]. Additionally, reactive oxygen species (ROS) signaling plays a vital role in regulating plant stress responses, with NADPH oxidases (NOXs) serving as key enzymes during salt stress [30]. Studies by Pilarska et al. [76] have shown that the expression patterns of two *NOX* genes, *RBOHD* and *RBOHF*, vary between salt-tolerant halophytes and more salt-sensitive glycophytes. In *Eutrema salsugineum*, the expression of these genes is induced by signals from abscisic acid (ABA) and ethephon, suggesting that a stable baseline of *NOX* activity in the leaves is essential for effective adaptation to saline environments [76].

## 4. Role of Transcription Factors in Salt Stress Response

Transcription factors (TFs) are crucial for modulating plant responses to environmental stress by precisely regulating gene expression. Mechanistically, TFs achieve this by binding to specific DNA motifs, usually in promoter or enhancer regions adjacent to target genes. The binding is mediated through the DNA-binding domain (DBD) of the TF, which selectively recognizes unique sequences in the DNA. Upon binding, TFs activate or repress transcription through their regulatory domains, which interact with the cell’s transcriptional machinery to initiate or block gene expression. This precise control allows plants to fine-tune cellular responses to environmental stresses, including salt stress, by quickly altering the expression of stress-response genes [79,80]. A key mechanism in salt stress tolerance involves the DREB (Dehydration-Responsive Element-Binding) transcription factor family, particularly the DREB2 subgroup. DREB TFs specifically bind to dehydration-responsive elements (DREs/CRTs) in the promoters of genes responsible for osmotic adjustment. This binding directly activates genes involved in osmolyte production, such as proline, which helps maintain cell turgor and protect cellular structures under saline conditions [81]. For example, in *Arabidopsis thaliana*, the overexpression of *DREB2A* upregulates these target genes, leading to enhanced osmoprotectant synthesis, improved water retention, and the stabilization of cellular proteins and membranes under high salinity. This increase in osmolyte levels translates to a 30% improvement in survival rates compared to wild-type plants, underscoring the effectiveness of DREB TFs in bolstering salt tolerance [82]. This mechanism exemplifies how TFs can activate stress-related pathways to improve plant resilience under saline conditions, thereby enhancing both survival and growth. Another important TF family is NAC (NAM, ATAF, and CUC), which regulates genes involved in ion transport, cell-wall biosynthesis, and detoxification processes. These functions are critical for maintaining cellular integrity under salt stress. A well-studied example is the *OsNAC6* gene in rice (*Oryza sativa*), which activates ion transporter and ROS detoxification genes under salinity conditions. Transgenic rice plants overexpressing *OsNAC6* displayed a 20% increase in grain yield under saline conditions compared to their non-transgenic counterparts, highlighting the potential of NAC TFs for enhancing salt tolerance [83]. The *WRKY* family is also integral to salt stress responses, particularly in mitigating oxidative damage. These TFs regulate the expression of genes encoding reactive oxygen species (ROS)-scavenging enzymes like superoxide dismutase (SOD) and catalase (CAT). For instance, the overexpression of *WRKY8* in Arabidopsis plants increased their salt tolerance by enhancing ROS detoxification [84]. This led to improved chlorophyll content and biomass production under high salt levels, showing the effectiveness of WRKY TFs in combating oxidative stress caused by salinity. The mechanisms of transcriptional regulation under salt stress involve multiple processes. TFs bind to specific cis-regulatory elements such as DRE or ABRE (abscisic acid-responsive element) in the promoters of salt-responsive genes. This interaction triggers the expression of genes involved in ion transport, osmoprotection, and oxidative stress responses [85]. Ion homeostasis is critical under salinity, with TFs like *NAC* and *WRKY* activating genes which manage the transport of Na^+^ and K^+^ ions to prevent Na^+^ toxicity. For example, *OsNAC6* upregulates the *HKT1* gene in rice, enhancing the plant’s ability to exclude sodium from its tissues and maintain a favorable K^+^/Na^+^ ratio [86]. Additionally, transcription factors regulate the scavenging of ROS, a harmful byproduct of salt stress. WRKY TFs, for instance, boost the production of ROS-scavenging enzymes, reducing oxidative damage. Moreover, TFs also modulate hormonal signaling pathways, such as those involving abscisic acid (ABA). In response to salt stress, certain TFs, like those from the bZIP family, interact with ABA-responsive elements to regulate genes that control water retention and stomatal closure, thereby reducing water loss. Transgenic approaches targeting transcription factors have yielded promising results in improving salt tolerance in crops. Overexpression of the *DREB2A* gene in *Arabidopsis thaliana* has led to higher survival rates and better biomass production under saline conditions, demonstrating the potential of targeting DREB TFs [87]. Similarly, transgenic rice plants overexpressing *OsNAC6* showed a significant improvement in yield under salinity, with a 20% increase compared to control plants. Another example is *WRKY8* in Arabidopsis, where overexpression resulted in enhanced ROS detoxification and greater salt tolerance, as evidenced by improved growth and chlorophyll retention under high salinity levels [88]. In soybean (*Glycine max*), overexpression of the *GmDREB1* gene improved salt tolerance by regulating the genes related to ion homeostasis and osmoprotection [89]. Transgenic soybean plants exhibited a 15% increase in pod formation under saline conditions compared to wild-type plants. These studies underscore the effectiveness of targeting transcription factors in developing salt-tolerant crops, offering a sustainable solution for agriculture in saline environments. Table 2 presents various transcription factors crucial for regulating plant responses to salt stress.

## 5. Next-Generation Sequencing Technologies in Salt Stress Research

Next-generation sequencing (NGS) technologies have transformed plant biology by enabling the rapid and high-throughput sequencing of genomes, transcriptomes, and epigenomes. These advancements have significantly reduced the time and costs associated with sequencing, allowing researchers to investigate complex traits, such as salt tolerance, in much greater detail. NGS has become an essential tool for identifying the genetic and molecular factors that underpin a plant’s ability to withstand abiotic stresses like salinity [98]. Its capacity to generate large volumes of genomic, transcriptomic, and epigenomic data is particularly beneficial for stress studies, as these data are crucial for elucidating the intricate mechanisms of plant stress tolerance. For instance, high-throughput RNA sequencing (RNA-Seq) enables the analysis of global gene expression changes in response to salinity stress, helping identify key genes and pathways involved in salt tolerance, including those associated with ion transport, osmotic adjustment, and the management of reactive oxygen species (ROS). Furthermore, NGS facilitates the discovery of novel stress-responsive genes and the characterization of regulatory networks, including transcription factors, which mediate these responses. Through the application of NGS in quantitative trait locus (QTL) mapping, researchers can identify genetic variations linked to salt tolerance traits, thereby paving the way for marker-assisted breeding and genetic engineering approaches. Additionally, NGS is valuable for studying epigenetic modifications that occur under stress conditions, offering insights into how plants adapt to salinity at the molecular level. The comprehensive data generated through NGS not only deepen our understanding of the genetic basis of salt stress tolerance but also aid us in the development of resilient crop varieties capable of thriving in saline environments. This is supported by the fact that, with the advent of NGS, plant breeders and geneticists can now sequence entire plant genomes, perform comprehensive transcriptome analyses, and integrate data from multiple omics layers, providing a deeper understanding of the plant’s adaptive responses to stress. Genome sequencing and assembly through NGS platforms such as Illumina, PacBio, and Oxford Nanopore have facilitated the identification of salt-tolerant genes and alleles [99]. By sequencing and assembling the genomes of salt-tolerant and salt-sensitive plant varieties, researchers can compare genetic variations between them. This has led to the discovery of single-nucleotide polymorphisms (SNPs), insertions/deletions (InDels), and structural variants associated with salt tolerance. For example, in *Oryza sativa* (rice), the sequencing and comparison of salt-tolerant varieties such as Pokkali with sensitive ones have uncovered alleles related to ion transporters like HKT1;5, which plays a critical role in sodium exclusion [100]. These discoveries provide valuable genetic targets for breeding programs and genetic engineering aimed at improving salt tolerance in rice and other crops. Transcriptome analysis using RNA sequencing (RNA-seq) is another powerful application of NGS that enables researchers to investigate gene expression patterns under different conditions, including salt stress. RNA-seq generates a comprehensive profile of all the transcripts expressed in a particular tissue at a given time, allowing for the identification of differentially expressed genes (*DEGs*) in response to salinity [101]. For instance, RNA-seq studies in *Arabidopsis thaliana* have identified thousands of *DEGs* under salt stress, including those involved in osmoprotection, ion transport, and ROS scavenging [102]. One significant discovery involved the overexpression of *SOS1*, a sodium–proton antiporter gene, which plays a crucial role in maintaining ion homeostasis under saline conditions. This information is critical for identifying candidate genes for genetic manipulation and selective breeding to enhance salt tolerance. Genome-wide association studies (GWASs) and quantitative trait locus (QTL) mapping, both of which leverage NGS, have been instrumental in identifying genetic loci linked to salt tolerance. GWAS is a population-based approach that scans the genomes of genetically diverse populations to find associations between genetic variants and phenotypic traits such as salt tolerance. In a GWAS study conducted on durum wheat (*Triticum durum*), researchers identified several SNPs associated with traits like shoot ion content and grain yield under saline conditions, providing valuable markers for marker-assisted selection (MAS) [103]. QTL mapping, on the other hand, helps to locate specific genomic regions that contribute to variation in salt tolerance traits using segregating populations derived from crosses between tolerant and sensitive lines. For example, in barley (*Hordeum vulgare*), QTLs associated with Na^+^ exclusion were mapped, leading to the identification of potential genes which contribute to salinity tolerance, thus offering new targets for breeding programs [104]. The integration of multi-omics data, including genomics, transcriptomics, proteomics, and metabolomics, represents a system biology approach to understanding plants’ response to salt stress at multiple biological levels. By combining data from these diverse omics platforms, researchers can construct comprehensive regulatory networks that govern plant responses to salinity. For example, a study in *Oryza sativa* integrated transcriptomic, proteomic, and metabolomic data to uncover a complex network of salt-responsive genes, proteins, and metabolites [105]. This system approach revealed key regulatory nodes, such as transcription factors (TFs) from the NAC and DREB families, which play a pivotal role in activating stress-responsive pathways. Furthermore, metabolites like proline and glycine betaine, which act as osmoprotectants, were shown to accumulate in response to salt stress, linking gene expression changes to physiological adaptations. These findings provide a holistic view of the salt stress response, offering new avenues for crop improvement through the targeted manipulation of key regulatory elements [106]. NGS technologies have fundamentally transformed our understanding of salt stress responses in plants. By enabling genome sequencing and assembly, transcriptome analysis, and the integration of multi-omics data, NGS provides valuable insights into the genetic and molecular mechanisms underlying salt tolerance. The application of GWAS and QTL mapping has further expanded the toolkit for identifying genetic loci associated with salt tolerance, paving the way for precision breeding and genetic engineering efforts to develop crops which are more resilient to salinity. Integrating genomics and transcriptomics provides a comprehensive understanding of how plants respond to salinity, revealing significant alterations in gene expression. For instance, Ouertani et al. [31] conducted a transcriptomic analysis on salt stress-responsive genes in barley (*Hordeum vulgare*), identifying substantial changes in gene expression: 3585 genes were upregulated and 5586 downregulated in the leaves, while, in the roots, 13,200 genes were upregulated and 10,575 were downregulated. This response includes mechanisms such as sensory and signaling pathways, transcriptional adjustments, hormonal regulation, osmoregulation, ion balance, and enhanced ROS scavenging, highlighting critical genes involved in hormone and kinase signaling, transcription factors, and transporters [31]. In addition to transcriptomics, proteomic and metabolomic studies are essential for understanding plant adaptation to salinity. Gan et al. [107] performed a comparative proteomic analysis of salt-tolerant and -sensitive mulberry varieties, identifying phenylpropanoid biosynthesis as a key factor in salt tolerance, which clarifies the molecular mechanisms behind mulberry’s resilience to saline conditions. Similarly, He et al. [108] discovered the *bolTLP1* gene in broccoli (*Brassica oleracea* var. *Italica)*, a thaumatin-like protein which enhances salt tolerance by modulating phytohormone signaling, enzyme activities, sulfur compound synthesis, and histone variant expression [85]. Further research has expanded our understanding of salt tolerance through genome-wide studies. Wang et al. [109] identified 60 genes in the cation proton antiporter (CPA) gene family in radish (*Raphanus sativus*), contributing to knowledge regarding salt tolerance in this species [86]. Chen et al. [110] explored the *CBL* gene family in apple (*Malus domestica*), revealing that the *Mdcbl10.1* gene positively impacts salt tolerance. Shao et al. [111] analyzed the wheat *14-3-3* gene family and found 17 potential genes with significantly reduced expression under alkaline stress, providing insights into their role in salt stress responses. Tan et al. [112] investigated the role of melatonin in apple plants, showing that transgenic lines with higher melatonin levels experienced less salt damage, reduced electrolyte leakage, and minimized chlorophyll loss compared to wild-type plants. These lines also had lower ROS levels due to increased antioxidant enzyme activity and the downregulated expression of the ABA synthesis gene *MdNCED3*. Zhang et al. [113] characterized the *PsnNAC036* gene in *Populus simonii* × *P. nigra*, demonstrating that its overexpression enhances both salinity and heat tolerance, promoting plant growth and stress resilience. NGS technologies have facilitated the study of genes like *BADH1*, with Min et al. [114] using haplotype analysis to explore its role in salt tolerance during rice domestication. Katja et al. [115] identified the jacalin-related lectin HvHorcH protein in root extracellular fluid, suggesting its potential role in plant adaptation to salinity. Yu et al. [116] discovered that the C2H2-type zinc-finger protein MpZFP1 from Millettia pinnata enhances salt tolerance in transgenic Arabidopsis by activating gene expression and scavenging ROS, resulting in improved seed germination and biomass accumulation. Additionally, Chun et al. [117] investigated the role of microtubule dynamics in salt stress response, finding that regulating microtubule-related genes could effectively enhance salt tolerance in crops. This integration of genomics, transcriptomics, proteomics, and metabolomics provides a holistic view of plant responses to salt stress, revealing key molecular and genetic factors critical for improving salt tolerance.

## 6. Challenges and Future Perspectives

While substantial progress has been made in understanding the genetic basis of salt tolerance through the study of transcription factors (TFs) and the application of next-generation sequencing (NGS) technologies, several challenges persist in translating this knowledge into practical solutions for agriculture. One significant limitation is the polygenic nature of salt tolerance. The trait is governed by multiple genes that interact in complex ways, often involving cross-talk between various signaling pathways. This complexity, coupled with the influence of environmental variables such as temperature, soil type, and water availability, makes it difficult to pinpoint all the genes involved in salt stress responses [57]. For instance, while TFs like *DREB* and *NAC* are known to activate stress-responsive genes, their regulatory networks are vast and not fully elucidated. In *Arabidopsis thaliana*, overexpression of the *DREB2A* gene improved salt tolerance under controlled conditions, but these benefits were not as pronounced in field trials, where additional environmental factors came into play [118]. Moreover, the high cost and technical expertise required for NGS technologies limit their widespread application, especially in developing countries. Although the cost of sequencing has dropped significantly, large-scale applications for breeding programs or genomic studies are still financially prohibitive for many institutions. This hampers the global adoption of advanced molecular breeding techniques [119]. Another set of challenges arises from ethical and regulatory considerations in developing genetically modified (GM) crops for salt tolerance. While GM crops hold great promise, public concern regarding their safety, ecological impact, and long-term sustainability remains high. In particular, worries about the unintended consequences of introducing GM crops into natural ecosystems, such as gene flow to wild relatives, potential effects on biodiversity, or the creation of "superweeds," complicate their acceptance, as detailed previously [120]. For example, the development of salt-tolerant GM crops like Bt rice has been met with regulatory hurdles and public opposition in countries like India and the Philippines, where activists have raised concerns about the safety and environmental implications of GM organisms [121]. Furthermore, regulatory frameworks governing GM crops vary widely across regions. In the European Union, strict regulations require extensive safety testing and risk assessments before GM crops can be approved, whereas other countries, such as the United States, have more lenient regulations. This disparity creates uncertainty for researchers and developers, as the time and cost required to navigate these regulatory systems can delay or halt the deployment of GM crops. Looking to the future, integrating advanced technologies such as CRISPR/Cas9, synthetic biology, and system biology presents promising avenues for overcoming current limitations in salt stress tolerance research. CRISPR/Cas9, a genome-editing tool, allows for precise modifications to specific genes associated with salt tolerance [122,123,124,125]. For example, CRISPR-mediated editing of the *OsHKT1;5* gene in rice has led to enhanced sodium exclusion and improved salt tolerance, showing the potential for CRISPR to accelerate the development of salt-tolerant crops [126]. Unlike traditional transgenic approaches, CRISPR/Cas9 offers the possibility of editing native genes without introducing foreign DNA, potentially alleviating some ethical concerns associated with GM crops. Synthetic biology could further expand possibilities by designing genetic circuits that fine-tune stress responses. For instance, custom-built pathways could be engineered to dynamically regulate osmoprotectant synthesis or ion transport under fluctuating salt conditions [127]. Additionally, system biology, which integrates data from genomics, transcriptomics, proteomics, and metabolomics, can help elucidate the intricate regulatory networks involved in salt stress responses. By combining omics data, researchers can build comprehensive models that identify key regulatory nodes, offering new targets for genetic manipulation and crop improvement. The development of salt-tolerant crops is particularly important for sustainable agriculture in saline environments. As soil salinization affects more than 20% of irrigated land globally, cultivating salt-tolerant crops has the potential to make previously marginal lands arable, reducing the pressure on freshwater resources and improving food security in areas prone to soil degradation [128]. For example, the introduction of salt-tolerant rice varieties like Pokkali in coastal regions of South Asia has demonstrated the potential of using salt-tolerant crops to sustain agricultural productivity under saline conditions. These crops have shown resilience in soils with high salinity levels, maintaining yield where conventional varieties fail [129]. Furthermore, the ability to cultivate crops on saline lands reduces reliance on freshwater for irrigation, a critical factor in regions facing water scarcity due to climate change. As research advances, integrating genetic engineering, molecular breeding, and sustainable farming practices could help develop salt-tolerant crops that play a crucial role in global food security. By tapping into the potential of technologies like CRISPR/Cas9 and synthetic biology and addressing regulatory and ethical concerns, the scientific community can work toward creating robust, resilient crops that thrive under adverse conditions. These innovations will be essential for maintaining agricultural productivity in the face of growing environmental challenges, offering a path toward more sustainable agriculture in saline and marginal environments.

## 7. Conclusions

This review has explored the critical role of plant genetics, transcription factors, and next-generation sequencing technologies in enhancing crop resilience to salt stress. Key genes and pathways involved in salt tolerance have been identified, and the role of transcription factors in regulating stress responses has been highlighted. NGS technologies have revolutionized the study of salt stress by enabling comprehensive genome and transcriptome analyses, leading to the discovery of novel genes and regulatory networks. Addressing the complex challenge of salt stress requires a multidisciplinary approach that integrates plant genetics, molecular biology, bioinformatics, and agricultural sciences. Collaborative efforts between researchers, breeders, policymakers, and farmers are essential for developing and deploying salt-tolerant crops effectively. Such an approach will ensure that the advances in basic research translate into practical solutions for improving crop resilience and agricultural productivity in saline environments. The future of crop improvement for salt tolerance lies in the continued integration of cutting-edge technologies such as CRISPR/Cas9, synthetic biology, and system biology. These tools offer unprecedented opportunities to enhance crop resilience and address the challenges posed by soil salinity. However, success will depend not only on scientific innovation but also on addressing ethical, regulatory, and socio-economic considerations. By taking a holistic and responsible approach, we can develop salt-tolerant crops that contribute to sustainable agriculture and global food security in the face of a changing climate.

## Figures and Tables

**Table 1:** Impact of salt stress on plants, including physiological and biochemical effects, mechanisms of salt toxicity, and economic and ecological implications.

Aspect	Description	Examples/Impacts	References
Physiological Effects	Salt stress negatively affects various physiological processes, including water uptake, photosynthesis, stomatal conductance, and nutrient acquisition.	Water uptake: This is reduced due to osmotic stress, leading to dehydration and wilting. Photosynthesis: This decreases due to stomatal closure and a reduced chlorophyll content. Nutrient deficiency: Salt interferes with potassium and calcium absorption.	[43]
Biochemical Effects	Salt stress alters biochemical pathways, leading to the accumulation of osmolytes, the production of reactive oxygen species (ROS), and changes in metabolic activities.	Osmolyte accumulation: Plants produce compatible solutes like proline and glycine betaine to maintain osmotic balance. Increased ROS production: This causes oxidative damage to membranes, proteins, and the DNA, affecting plant metabolism and growth.	[61]
Ion Imbalance	High concentrations of sodium (Na^+^) and chloride (Cl^−^) ions disrupt nutrient ion balance (e.g., potassium and calcium), leading to toxicity and impaired cellular functions.	Sodium toxicity: Na^+^ competes with potassium (K^+^), disrupting enzymatic activities and leading to growth inhibition. Chloride toxicity: The accumulation of Cl^−^ affects nutrient transport and enzymatic functions in leaves.	[62]
Osmotic Stress	Excess salts in the soil lower the soil water potential, making it difficult for plants to absorb water, causing dehydration and wilting.	Reduced cell turgor: The loss of water uptake leads to cell shrinkage and impaired growth. Delayed germination: Seeds fail to germinate properly under high salinity due to the lack of water availability.	[63]
Oxidative Damage	Salt stress induces the production of ROS, leading to oxidative stress and damaging cellular structures, proteins, lipids, and the DNA.	Lipid peroxidation: ROS causes damage to membrane lipids, leading to the leakage of ions and the loss of cellular integrity. Protein denaturation: ROS-induced damage disrupts enzymatic activities and photosynthetic machinery.	[64]
Economic Implications	Salt stress reduces crop yields and quality, leading to economic losses in agriculture, particularly in regions dependent on irrigated farming.	Yield reduction: Crops like rice, wheat, and maize exhibit up to 50% yield losses in highly saline environments. Economic losses: Globally, salinity is responsible for billions of dollars in crop production losses each year.	[65]
Ecological Implications	Soil salinization affects biodiversity, soil health, and ecosystem functions, making large areas of land unproductive.	Land degradation: Over 20% of irrigated land worldwide is affected by salinity, reducing arable land availability. Ecosystem disruption: High salinity leads to the loss of soil microbial diversity and negatively impacts freshwater ecosystems.	[66]

**Table 2:** Various transcription factors that play pivotal roles in modulating plants’ response to salt stress, emphasizing their regulatory functions in different stress response pathways, such as osmotic adjustment, ion transport, and oxidative damage mitigation.

Transcription Factor (TF)	Role in Salt Stress Response	Impacts	References
DREB (Dehydration-Responsive Element-Binding Protein)	Regulates gene expression in response to abiotic stresses like drought, salinity, and cold by activating stress-responsive genes in the abscisic acid (ABA)-independent pathway.	DREB1A overexpression in wheat: Enhances salt tolerance by improving water retention and ion homeostasis under saline conditions. DREB2A in Arabidopsis: Activates stress-responsive genes related to osmotic adjustment and salt tolerance.	[90]
NAC (NAM, ATAF1/2, and CUC2)	Regulates stress-responsive genes that control plant growth, development, and stress responses, especially under high-salinity conditions.	SNAC1 in rice: Enhances drought and salt tolerance by regulating stomatal conductance and reducing water loss. NAC57 in Arabidopsis: Controls genes associated with cell-wall integrity and stress signaling pathways.	[91]
WRKY	Modulates the expression of stress-responsive genes involved in abiotic stress tolerance, especially in oxidative stress and hormone signaling pathways.	WRKY46 in rice: Overexpression enhances salt tolerance by regulating antioxidant systems and stress-related gene expression. WRKY18 in Arabidopsis: Regulates genes involved in salinity tolerance and ROS detoxification.	[92]
MYB (Myeloblastosis)	Regulates the expression of genes involved in secondary metabolism, cell-wall biosynthesis, and abiotic stress responses such as drought and salinity.	MYB96 in Arabidopsis: Controls salt tolerance by activating ABA signaling and promoting proline accumulation, which helps in osmotic adjustment. MYB44 in rice: Improves salt and drought tolerance by enhancing ion transport and antioxidant defense.	[93]
bZIP (Basic Leucine Zipper)	Plays a role in ABA signaling pathways under stress conditions, regulating genes involved in osmotic adjustment and ion transport.	bZIP17 in Arabidopsis: Functions in the endoplasmic reticulum (ER)’s stress response, improving plant tolerance to salt stress by regulating ion transporters and stress-related genes.	[94]
AP2/ERF (APETALA2/Ethylene-Responsive Factor)	Regulates stress-responsive genes, particularly those related to ethylene signaling and osmotic stress responses, including ion transport and detoxification.	ERF1 in Arabidopsis: Increases salt tolerance by regulating ion transport and antioxidant defense. ERF5 in tomato: Enhances salt tolerance by activating the genes involved in ion homeostasis and reducing Na^+^ accumulation in tissues.	[95]
HD-Zip (Homeodomain-Leucine Zipper)	Regulates stress responses, including salt stress, by controlling developmental processes, hormone signaling, and abiotic stress tolerance mechanisms.	HD-Zip1 in rice: Improves salt tolerance by enhancing ABA-mediated responses and ion transport under saline conditions. ATHB-7 in Arabidopsis: Functions in salt and drought tolerance through the regulation of stress-responsive genes.	[96,97]

## Data Availability

Not applicable.
